# NEWS

**DOI:** 10.7189/jogh.06.020202

**Published:** 2016-12

## The Bill and Melinda Gates Foundation

➢ Bill Gates expressed support for the potential of genetic engineering to eliminate the species of mosquitoes which spread malaria and dengue fever. The CRISPR/Cas9 gene from bacteria could be used to edit the mosquitoes’ DNA to prevent them from reproducing, causing that particular species to become extinct. Bill Gates noted that the malaria– and dengue–carrying mosquito species are a small percentage of mosquitoes, and eliminating them would not endanger the environment or harm people. In April, he called these mosquitoes “the most dangerous animal in the world”, and does not share the belief that the technology could be a “weapon of mass destruction”. (*Forbes*, 10 June 2016)

➢ In a blog post, Bill Gates highlighted how raising chickens is an easy and cheap way to ensure a food supply and earn an income for poor people with access to land. Chickens are cheap to maintain, as they can often live on food they find themselves, and only need basic shelters to protect their nests and eggs. Chickens stay close to home, making them easier to look after. A farmer with 5 hens which are fertilised with a neighbor’s rooster can produce 40 chicks within three months. In West Africa, these chicks can sell for US$ 5 apiece, generating up to US$ 1000 a year. This is a small but significant step–up from the absolute poverty–line of US$ 700 a year. Lastly, eggs are also a protein–rich foodstuff. (*Fortune*, 8 June 2016)

➢ Through a project financed by the BMGF among other partners, Uganda is planting five new bean varieties, which are both high in iron and drought–resilient. The beans – a cheap source of nutrition for poorer communities – are part of a wider effort to reduce malnutrition and reduce anemia, particularly among children and pregnant women. Iron deficiency is a major nutritional problem affecting 2 billion people worldwide which can impair children’s cognitive and physical development, and anemia makes pregnancy and childbirth more dangerous to women. The beans were co–developed with the Rwandan Agricultural Board through the HarvestPlus program, using iron sources with locally–adapted germplasm in Uganda. The improved yields will also boost farmers’ incomes, and the beans’ drought resilience is vital for small–scale farmers and communities. (*Thomson Reuters Foundation*, 10 August 2016)

➢ The BMGF has supported new research into the causes of childhood diarrhea, which kills 500 000 children each year. The study, led by the University of Virginia School of Medicine (Division of Infectious Diseases and International Health), looked at diarrhea cases in Bangladesh, India, Pakistan, the Gambia, Kenya, Mali and Mozambique. The researchers re–analyzed more than 10 000 archived samples from the Global Enteric Multicenter Study (GEMS), and found two more causes of diarrhea: *C. jejuni* and adenovirus, and that the number of cases caused by *Shigella,* adenovirus, *Camplyobacter* and *E coli* was significantly underestimated. This means that many unexplained diarrhea cases are caused by a known rather than an unknown pathogen, thus giving a roadmap for treating childhood diarrhea as it is more feasible to develop vaccines and treatments for a smaller number of known pathogens. “For the first time in history, we can identify almost all of the pathogens that cause diarrhoea, thanks to the GEMS re–analysis study. And now that we know more, we can more precisely target our efforts to make sure every child has the opportunity to live a healthy and productive life,” said Anita Zaidi, director of the BMGF’s Enteric and Diarrheal Diseases team. (*Medical Xpress*, 27 September 2016)

➢ The BMGF is working to provide more phones and financial services to women around the world, highlighting that 1.7 billion women in low– and middle–income countries don’t have access to mobile phones – more than 400 million fewer women than men in these countries. Mobile phones are bringing down the cost of providing basic financial services, but the technological gender gap means that those outside the formal banking system who would benefit most from easier access to savings, small loans and money–transfer facilities can’t be reached if they don’t have mobile phones. More than 50% of this gender gap occurs in India, where women are often prevented from accessing technology by fathers and husbands, and only 30% of internet uses in India are female. Financial inclusion in developing countries like India is particularly important, as women are more likely than men to use financial services and more likely to use them in a way that will benefit their families. The BMGF is working with mobile operators and the GMSA [a global association of mobile phone providers] Mobile Money Programme, to bring more mobile money services to those outside the formal banking system. (*Wall Street Journal*, 26 October 2016)

## The GAVI Alliance

➢ Diarrhea caused by rotavirus is responsible for 33% of hospitalisation among Myanmar’s children. In response, health officials are calling for the rotavirus vaccine to be added to the country’s National Immunisation Program. They acknowledge that the government cannot afford to include it, but are campaigning for an application to GAVI to support its introduction, which is currently only available privately. GAVI currently supports 5 vaccine programs in Myanmar, including measles and pneumococcal immunisations. Children who are malnourished, or with impaired immunity are more vulnerable to the virus. A spokesperson for Myanmar’s Ministry of Health and Sports confirmed that they plan to implement the rotavirus vaccine with support from GAVI and UNICEF. (*Myanmar Times*, 22 June 2016)

➢ GAVI has pledged US$27.5 million to a WHO pilot project on the introduction of the malaria candidate vaccine RTS,S in sub–Saharan Africa. GlaxoSmithKline, the vaccine developer, and PATH will donate vaccine doses in the pilot areas. In addition to GAVI funding, the WHO will provide US$ 17 million and PATH has received US$ 8 million from the BMGF for the project. GAVI funding is conditional upon the WHO securing additional funding to cover the remaining shortfall – an estimated US$ 27.5 million. RTS,S was developed as a “not–for–profit” vaccine with shared costs, and GlaxoSmithKline has invested more than US$ 350 million in its development. David Kaslow, the Head of PATH’s Center for Vaccine Innovation and Access, said that the results from the Phase 3 studies showed the potential benefit of RTS,S, when used alongside bed–nets and other malaria control interventions. (*Regulatory Affairs Professional Society*, 23 June 2016)

➢ GAVI and health technology company Royal Philips have signed a letter of intent to jointly develop scalable digital transformation plans to improve the quality of immunisation data, and its collection in primary and community care. This will help countries improve the planning, coverage and impact of their immunisation programmes, by identifying children who miss out on vaccination. “We are convinced that partnerships, such as this one with GAVI, are paramount to realising the goal of universal health coverage for all, especially among children. Thanks to the digital revolution, we can now start doing what was previously unthinkable: enable access to care, improve patient outcomes and lower healthcare costs,” says Jeroen Tas, CEO of Connected Care and Health Informatics at Phillips. This partnership is one example of how GAVI aims to step up its data–strengthening activities and address some of the fundamental weaknesses in data collection systems in the countries which it supports. (*ReliefWeb*, 21 September 2016)

➢ Médecins Sans Frontières (MSF) has declined a donation from Pfizer of 1 million pneumonia vaccines which would protect against a leading killer of children. Instead, MSF argues that the donation would harm the long–term aim of lower prices for vaccines, and that accepting it – which would save lives – involves a trade–off by reducing access to affordable vaccines and medicines. GlaxoSmithKline, the only other producer of the pneumonia vaccine, recently lowered its price to US$ 3.05 per dose for humanitarian organisations, but Pfizer only offers discounted prices to GAVI while offering free donations to humanitarian organisations. Vaccine prices are increasing, and it is now 68 times more expensive for MSF to fully vaccinate a child in 2016 compared to 2001, and 45% of the cost is attributable to pneumonia vaccines – leading to MSF to call for the price of pneumonia vaccines to be reduced to US$ 5 per child. MSF argue that countries currently eligible for GAVI support will start losing it in 2016, citing Angola which will shortly have to buy pneumonia vaccines itself, leading to a 1500% price increase per dose; and Bolivia and Indonesia will also see massive price increases. Pfizer made US$ 13.1 billion in profits in 2015, and sales of pneumonia vaccine in developed countries forms a large chunk of its profits, so offering cheaper prices to humanitarian organization and developing countries may not significantly reduce its profits. Pfizer believes that donations have a crucial role in addressing humanitarian crises, but MSF argue that donations remove incentives for new manufacturers to enter a market, thereby reducing competition and keeping prices higher. (*Humanosphere*, 25 October 2016)

➢ In the wake of Hurricane Matthew, large teams are being mobilised by the Haitian Ministry of Health and Population to vaccinate 800 000 people in the worst–affected areas against cholera. This initiative is being supported by the PAHO and WHO, with vaccines supplied by GAVI. It aims to reduce morbidity and mortality from cholera, and to prevent its spread to other areas of the country. Vaccination will offer protection against 60–70% of severe cases, but other measures, such as daily chlorination of water, hand–washing, food hygiene, drinking potable water, and rehydration and treatment for diarrhea are also vital. Since Hurrican Matthew struck Haiti on 4 October 2016, there has been a significant increase in the number of suspected cases and deaths from cholera. (*ReliefWeb*, 9 November 2016)

## The World Bank

➢ The World Bank has approved a loan of US$ 310 million to help build climate resilience in Vietnam and ensure sustainable livelihoods for 1.2 million people in the Mekong Delta region. The Mekong Delta supports 50% of Vietnam’s rice production, 70% of its aquaculture and 30% of GDP. However, recent extreme weather in this area, including drought and salinity intrusion, is having an adverse impact on the many poor farmers living in the area, and it is extremely vulnerable to climate change as well as upstream development. The funding will help farmers adapt agriculture and aquaculture to climate change, including better planning, and land and water management. The World Bank will work in partnership with the government to deliver the project, plus other key development partners. (*thanhniennews.com*, 13 June 2016)

➢ The World Bank has approved funding of US$ 200 million toward social safety programmes implemented by the Tanzania Social Action Fund (TASAF) – more than 6.6 million Tanzanian citizens (15% of the country’s population) who live in extreme poverty or food security will benefit. Tanzania’s Productive Social Safety Nets (PSSN) project, which funds TASAF, will support the government’s recent scaling–up of conditional cash transfers and the increased participation of key beneficiaries in new programmes of public infrastructure, savings and investments. The PSSN has already reached the poorest 15% of Tanzania’s population, and the additional funding will consolidate its impact. PSSN is part of a wider World Bank Group project, which targets people living in extreme poverty to create human capital to reduce poverty and inequality. (*allafrica.com*, 18 June 2016)

➢ The World Bank and Global Fund have committed to invest US$ 24 billion in Africa over the next 3–5 years to support universal health care, and to help countries put surveillance systems in place for early disease detection. At the same time, Kenya pledged to increase its Global Fund contribution from US$ 2 million to US$ 5 million. According to the WHO, each year 100 million fall into poverty because of health care expenses, and 1 billion people cannot access health care. “African countries can become more competitive in the global economy by making several strategic investments, including investing more in people, their more prized resource. A critical part of this commitment is to accelerate progress on universal health coverage – ensuring that everyone, everywhere has the opportunity to live a healthy and productive life,” says Mr Jim Young Kim, President of the World Bank Group. (*Daily Nation*, 27 August 2016)

➢ According to the World Bank study *Poverty and Shared Prosperity,* the number of people living in extreme poverty continues to fall despite the 2008–09 financial crisis and slowing global economic growth. It shows that in 2013, fewer than 800 million people lived on less than US$ 1.90/day; this is 11% of the world’s population, compared to 35% in 1990 and means that 1.1 billion people have moved out of extreme poverty. Wages rose for the poorest 40% of people in 60 out of 83 countries studied between 2008 and 2013. The World Bank links future poverty reductions with falling inequality, noting that inequality has fallen in many countries over the past 10 years. Among countries which have reduced inequalities in recent years (eg, Brazil, Cambodia, Mali, Peru, Tanzania etc), the World Bank identified the importance of early childhood development and nutrition; universal health coverage; universal access to quality education; cash transfers to poor families; improved rural infrastructure; and progressive taxation. The main drivers in poverty reduction have been countries in East Asia and the Pacific, but as their poverty levels continue to fall, their contributions to poverty reduction will fall too. It also means that 50% of extremely poor people now live in sub–Saharan Africa, and that the remaining pockets of poverty will become harder to reach and address. (*NPR*, 3 October 2016)

➢ The remits of multilateral development banks (MDB) – including the World Bank – have not changed in line with challenges presented by the 21st century, according to a report published by the Center for Global Development. These challenges include: climate change, which will require more investment, especially global private capital, in greener infrastructure; the health risks posed by growing resistance to antibiotics and the possibility of pandemics; and helping the millions of refugees from conflicts such as the Syrian and South Sudan civil wars require investment in education and jobs for the displaced people. However, MDBs rely heavily on country–based loans to achieve these aims, which can be an inflexible and often inappropriate instrument in today’s climate. MDBs can be held up by unfulfilled donor pledges and debates on what constitutes a poor country; but to respond quickly to a pandemic or refugee crisis they need dedicated contingency funds and more innovative financing. Currently, these are addressed through small special funds and one–off budgetary set–asides. The MDBs not only need more resources, but should unlock their existing assets and capital resources and use them more flexibly, and developing countries – now over 50% of the global economy – should be adequately represented in decisions about investment and sustainability. (*Bloomberg View*, 6 October 2016)

## UNAIDS and The Global Fund

➢ UNAIDS announced that from 2010 to 2015, the number of HIV–positive people taking antiretroviral therapy (ART) has more than doubled to 17 million people, with 2 million people gaining access to treatment in 2015 alone. Global ART coverage has reached 46%, and coverage in eastern and southern Africa – the areas most affected by HIV – increased from 24% to 54%, reaching 10.3 million people. The ART scale–up has reduced AIDS–related deaths from 1.5 million in 2010 to 1.1 million in 2015. However, these figures mask regional disparities, and falling rates of new HIV infections in young adults have begun to slow, with the estimated number of new infections almost static. The world must continue this momentum in order to reach the 90–90–90 goal (90% of HIV–positive people know their status, 90% receive treatment, and 90% of people on treatment have viral suppression) by 2020, and to be on track to end the AIDS epidemic by 2030. (*allafrica.com*, 1 June 2016)

➢ At a workshop in Thimphu, Bhutan, the health secretary Dr Dorji Wangchunk highlighted how Global Fund support for dealing with HIV, tuberculosis (TB) and malaria has enabled Bhutan to work toward meeting the Millennium Development Goals. To date, the Global Fund has allocated more than US$ 13 million (US$ 4.1 million for HIV/AIDS, US$ 3.6 million for TB and US$ 5.4 million for malaria). Bhutan has moved from the “least development category”, and some donors have begun to phase out support. Although the Global Fund has placed Bhutan in the “pre–elimination” phase, it is still eligible for funding after the end of the New Funding Model in 2018, but despite this continuation overall external support is decreasing from 30% of expenditure in the 1990s to 12% today. This means that alternative mechanisms, such as the Bhutan Health Trust Fund (BHTF) are needed, to provide co–financing for sustaining public–health programmes. Bhutan’s successes in treating these conditions are illustrated by 160 people with HIV accessing antiretroviral treatment, 4200 TB cases have been diagnosed and treated, and 362 000 insecticide–treated bed–nets. (*kuenseloline.com*, 9 June 2016)

➢ Jamaica has seen real gains in the fight against HIV/AIDS, such as a mother–to–child transmission rate under 2%, and sharp falls in the prevalence rate, including among children and sex workers. However, there are concerns that these gains could be reversed as the Global Fund transitions its support to Jamaica in 2018, due to its attaining middle–income status. In 2010, the Global Fund awarded Jamaica US$ 40 million, but in 2015 this fell to US$ 15 million, and the funding cycle moved from 5 to 3 years. It is anticipated that Jamaica may transition out of receiving Global Fund support after this current funding round. With reduced support, Jamaica is focusing on key populations, eg, people living with HIV, men who have sex with men, transgender people and sex workers. There are concerns that the Jamaican government will not be able to cover the funding shortfall following the withdrawal of Global Fund support. This may compromise the recent government’s declaration to fast–track efforts on the fight against HIV/AIDS, and to end the AIDS epidemic by 2030. “Civil societies are now looking at their own transition plan. We are praying that it [Global Fund] doesn’t go. We are advocating that it doesn’t go,” says Kandasi Levermore of the Jamaica AIDS Support for Life organization. (*Jamaica Gleaner*, 26 June 2016)

➢ Global Fund supporters, including governments, the private sector and civil society partners, have pledged US$ 12.9 billion for the 5th Voluntary Replenishment of the Global Fund to Fight AIDS, Tuberculosis and Malaria. However, the Global Fund’s investment case states that this falls short of the amount needed to meet the 2030 global targets for these diseases; and that US$ 13 billion is 80% of the amount needed. The shortfall is expected to be met by partner countries, but despite growing health expenditure, it is insufficient to bridge the gap. If the world meets the global 2020 target of mobilizing US$ 26 billion per year for the HIV/AIDS response in low–and middle–income countries, it will achieve a 15–fold return on investment, and in the long–term it will save money by preventing 21 million AIDS–related deaths and 28 million new HIV infections. (*ReliefWeb*, 21 October 2016)

➢ The Global Fund and partners are working together to support countries which are experiencing problems in implementing grants. These problems can include grants starting late, falling behind schedule, and difficulty absorbing all the financing they have been awarded. This initiative, known as Implementation Through Partnership (ITP) aims to alleviate bottlenecks, increase efficiency and effectiveness, and thereby maximising impact. It is focusing on the 20 countries which received more than US$ 150 million in Global Fund allocations, but have expenditure vs budget rates of under 70%, or require 50% or more of expenditure scale–up, or have more than 20% of their grant allocations forecast to be undispersed. These countries, which include Benin, Ghana, DR Congo, India and Pakistan, will receive technical support, leadership and advocacy to launch projects, and evolve from “implementation” to “impact”. Post ITP, the Global Fund plans to learn from what worked well and integrate them into a sustainable model for future grants. (*aidspan*, 1 November 2016)

## United Nations

➢ The UN Security Council adopted a resolution that allowed the interception of ships at sea destined for Libya, in an effort to target the flow of weapons into the country, where Islamic State is gaining ground. It authorises military intervention to inspect vessels going to or from Libya, and seizing any weapons on board. Arms embargoes have been in place since 2011, but are ineffective in blocking weapons from countries such as Egypt, Turkey and the United Arab Emirates. Libya has been in disarray since the fall of Moammar Gadhafi, and the lack of a strong government and state institutions have created a breeding–ground for terrorist networks like Islamic State and Al Qaeda. The UN–brokered national unity government (the Government of National Accord) is struggling to gain control, and the continued instability is a major factor behind the high numbers of people fleeing to Europe across the Mediterranean Sea. France’s ambassador, François Delattre, said that the resolution was a “game–changer” as it finally gives the UN and EU the means to enforce the arms embargo, and be strengthened in the fight against Islamic State. (*Wall Street Journal*, 14 June 2016)

➢ A UN report on poverty in Latin America found that the region grew more equal, with nearly 50% of its population enjoying increased income and 25% joining the middle class between 2003 and 2013. This is coupled with only 1% moving into a lower–income group, and the share of people living on less than US$ 2.50 a day fell by half, to 11.5%. This has led to Latin America’s Gini coefficient [an indicator of inequality] falling from 0.55 in 1994 to 0.49 in 2013. However, less favorable economic conditions due to the end of the global commodity boom have caused the region’s GDP growth to falter at 0.6% per annum. Income growth among poorer people is especially vulnerable to economic shocks, and the report states that without robust economic growth, poverty reduction programmes (eg, cash transfers) may be insufficient to prevent people falling back into poverty. Downward mobility can be cushioned by more secure jobs with benefits, assets, help with caring for dependents, and formal safety nets such as unemployment payments and pensions, but these indicators all appear troubling for Latin America, and its success in poverty reduction may be as fleeting as the commodity boom which made it possible. (*Economist*, 18 June 2016)

➢ The UN may admit a role in the outbreak of cholera in Haiti [following the 2010 earthquake] which killed at least 10 000 people and sickened hundreds of thousands of others. Although the potential admission stops short of admitting that the UN caused the epidemic, and does not indicate any changes to its immunity from legal actions, it nonetheless represents a significant shift in the UN’s position after five years of denial of any responsibility. The outbreak appears to have originated from a base housing UN peacekeepers recently returned from Nepal, where a cholera outbreak was under way – the base’s effluence was discharged into a nearby river. Mr Philip Alston, a UN special rapporteur who wrote a confidential report which states that “the epidemic would not have broken out but for the actions of the United Nations”, has criticised the UN’s avoidance of acknowledging the outbreak’s source, and states that the UN’s overall credibility and integrity is undermined by its actions. He also critises the UN’s cholera eradication program in Haiti, claiming it has failed – infection rates have risen since 2014 and the UN has struggled to raise the US$ 2.27 billion needed to eradicate cholera in Haiti. No major water or sanitation projects have been completed, and two pilot projects were closed due to a shortage of funds. A separate UN report shows that 25% of UN sites in Haiti were still discharging waste into public canals as late as 2014. The Second Circuit Court of Appeals in New York is considering a decision on a case against the UN brought by families affected by the cholera outbreak – the UN has previously claimed diplomatic immunity. (*New York Times*, 17 August 2016)

➢ In an unusual move, the UN General Assembly, which rarely discusses health–related issues outwith crises, will debate the growing threat of bacteria which are resistant to antibiotics. This is a recognition that antimicrobial resistance (AMR) must be addressed by world leaders, and a UK government report has estimated that deaths from AMR could rise from 700 000 a year to 10 million a year by 2050, costing US$ 1 trillion in lost production. AMR is caused by a number of factors, such as people taking antibiotics incorrectly or stopping them too soon, and the massive and often inappropriate use of antibiotics in agriculture, where antibiotics are used to promote growth and deal with infections among farm livestock. Countries will need to develop action plans to deal with AMR, although many member states have failed to deliver on promises to tackle AMR. (*Business Insider*, 19 September 2016)

➢ According to UN envoy Staffan de Mistura, rebel–held eastern parts of the Syrian city of Aleppo may face “total destruction” within two months, with thousands of deaths. The city of 275 000 people has been besieged for a month, as Russian and Syrian forces attack the jihadist group Jabhat Fateh al–Sham following the breakdown of a ceasefire in September 2016. At least 200 wounded civilians are in need of evacuation to save their lives, and at least 376 people have been killed and 1266 wounded over the past 2 weeks. Mr de Mistura offered to personally accompany jihadists linked to al–Qaeda out of the city if it would stop the fighting, and appealed to Russia and Syria’s government not to destroy the city in order to eliminate rebels, warning that history would judge them if the used the presence of jihadists in Aleppo as “an alibi perhaps for destroying the whole city.” (*BBC*, 6 October 2016)

## UNICEF

➢ According to UNICEF, 90% of the children who arrive in Italy from North Africa are not accompanied by adults, and 7009 unaccompanied children made the journey in the first 5 months of 2016 – twice the 2015 level. There are currently 235 000 refugees and migrants in Libya and 956 000 in the Sahel countries, and many, if not most, of these people will hope to cross to Europe. UNICEF is concerned about the number of children who do not register themselves when they arrive in Europe, but continue onwards and become vulnerable to criminal gangs. Italian social workers have found that boys and girls were sexually assaulted and forced into prostitution in Libya, and that some girls were pregnant on arrival, after being raped. Aimamo, a 16–year old boy, reported conditions of near–slavery on the farm in Libya where he and his brother worked to pay the people–smugglers. Marie–Pierre Poirier, UNICEF’s special co–ordinator for the European migrant crisis, says that these children have “endured war, persecution, deprivation and terrible journeys,” and that “even when they have reached the relative safety of their destination, they still need protection, education, healthcare and counselling. We must be on their side.” (*Al Jazeera*, 14 June 2016)

➢ UNICEF’s latest *State of the World’s Children* report shows that more than 69 million children will die from mostly–preventable diseases by 2030, and 167 million will be living in extreme poverty, unless world leaders take decisive action. 2030 is the target date for achieving the Sustainable Development Goals. Nearly 50% of these child deaths will be sub–Saharan Africa, where at least 247 million children – nearly 2–in–3 children – lack the necessary resources to survive and develop. UNICEF’s executive director, Anthony Lake, says that these children are mostly reachable, but there are political and resource constraints. Although the world has made huge progress in tackling child mortality, school enrolment and poverty reduction, the report highlights that the world’s most disadvantaged people are still missing out, as the poorest children are still twice as likely to die before their 5th birthday and suffer from chronic malnourishment, compared to their richer peers. This picture is even worse in sub–Saharan Africa, where children are 10 times more likely to die before their 5th birthday compared to children in richer countries, and by 2030 it will have 90% of the world’s poor children, and over 50% of the 60 million children who are not in school. One of the report’s authors, Kevin Watkins, says attention must be shifted to tackling child labour and early or forced marriage to improve schooling rates, and the onto needs of refugee children who are displaced and forced into poorly–paid jobs to support their families. The report calls for cash transfers to support children in school, among other measures. (*The Guardian*, 28 June 2016)

➢ UNICEF has confirmed that 145 child soldiers have been released following negotiations with two rebel groups – the Cobra Faction and the main SPLA/IO group – in South Sudan. Their liberation including disarming them, providing them with civilian clothes and enrolling them into a re–integration programmes, where they receive counselling and support with re–connecting with their families. UNICEF notes that at least 16 000 children remain fighting in the frontlines or working as porters with both armed militia and the national army, with 8000 children being recruited this year alone. The three–year conflict in South Sudan has killed thousands of people, and driven more than 2.5 million people from their homes, and the UN has called for an arms embargo to prevent further violence. UNICEF hopes that the latest release of these children will be followed by others, and UNICEF’s head in South Sudan, Mahimbo Mdoe, called on all parties to abide by international law, end the recruitment of child solders, and to release children who are currently serving. “Children in South Sudan need safety, protection and opportunities,” he said. (*New Arab*, 26 October 2016)

➢ Anthony Lake, the Executive Director of UNICEF, has classed the airstrikes on a Syrian school which killed an estimated 22 children and 6 teachers, as a “war crime”, if they were deliberate. He noted that they may be the deadliest attack on a school since the beginning of the war. The airstrikes took place in the rebel–held province of Idlib, which is the main Syrian opposition stronghold although radical militant groups also have a major presence. An eye–witness said that as many as 10 airstrikes may have hit the residential area where the school is located. Across Syria, UNICEF confirms that 591 children were killed in 2015 as a result of the civil war, 1.7 million children did not attend school, and another 2.1 million children are at risk of dropping out of school. In the besieged city of Aleppo, teachers and volunteers have set up some schools underground to ensure that some teaching can continue amidst the air–strikes. (*Sky News*, 27 October 2016)

➢ UNICEF has announced seed innovation seed funding of US$ 9 million, to enable 5 start–up companies to create affordable mobile connectivity, blockchain [a distributed database that maintains a continuously–growing list of records] in childhood development, data collection in maternal care, and technology to help improve literacy skills. This is the first time that UNICEF has funded start–up companies, and the 5 companies (SayCel, mPower Social Enterprises, 9 Needs Pvt, Innovations for Poverty Alleviation, and Chatterbox Dating Mobile), were chosen because they are building solutions to the “world’s most pressing problems”, accord to Chris Fabian of UNICEF’s Innovation Unit. The intellectual property of each company’s work will be made available under open source – one of UNICEF’s requirements for funding these companies. (*Wired*, 15 November 2016)

## World Health Organization (WHO)

➢ Amid a global vaccine shortage, the WHO has recommended cutting the standard dose of yellow fever vaccine by 80% (“fractional dosing”) in emergencies. Emergency stocks have been depleted by a mass immunisation drive in Angola, where more than 300 people have died from the disease since December 2015, and there has been a surge of cases in the DR Congo. The DR Congo has reported 1044 suspected cases of yellow fever since March 2016 and 71 deaths. The WHO cautioned that fractional dosing is a short–term measure for emergency situations where a shortage exists. The WHO maintains that its current vaccine levels are adequate, but an outbreak in the DR Congo’s capital city, Kinshasa, meant that fractional dosing is being seriously considered to “prevent transmission through large–scale vaccination campaigns.” Fractional dosing provide immunity for at least 12 months in healthy adults, although it is unclear if they are effective for young children. (*BBC*, 17 June 2016)

➢ The WHO announced that Thailand is the first country in Asia to eliminate the mother–to–child transmission of HIV and syphilis. Elimination means the reduction of transmission to such a low level that it is no longer a public health problem, and in Thailand this fell from 1000 children in 2000 to 85 children in 2015. Although Cuba is the first WHO–validated country to eliminate mother–to–child HIV transmission, Thailand – with 450 000 people living with HIV – is the first country with an HIV epidemic to do so. Thailand was affected by a massive HIV epidemic in the 1980s and 1990s, and had responded with awareness and condom campaigns and free antiretroviral treatment, which have cut the number of new infections from 143 000 in 1991 to 8100 in 2013. Thailand also provided all pregnant women – including undocumented migrant workers – free antenatal care, delivery and services for HIV and syphilis, which combined with scaled–up coverage rates has led to this success. According to Dr Poonam Khetrapal Singh, WHO’s regional head for Southeast Asia, “this is a remarkable achievement for a country where thousands of people live with HIV. Thailand has demonstrated to the world that HIV can be defeated.” (*Reuters*, 7 June 2016)

➢ The WHO says its staff have been obstructed from doing their work in South Sudan's capital and expressed concern about the conflict's likely impact on health care services. A WHO spokesman, Tarik Jašareviæ, said “the movement of WHO staff in Juba was being restricted by military forces”, but also that the WHO had supplied the Juba Teaching Hospital with essential medicine and body bags. “Medical kits would be distributed to partners on protection of civilians sites, and the WHO was mobilizing additional human and financial resources,” he said. Mr Jašareviæ also pointed outthat even before the latest fighting the health care sector faced funding shortfalls this year. “Out of the US$ 7.5 million which the WHO needed for health interventions in South Sudan, only US$ 4.3 million had been received thus far. The health cluster as a whole was only 28 percent funded,” he said. (*Radio Tamazuj*, 13 July 2016)

➢ According to the WHO, a global shortage of HIV testing could undermine efforts to diagnose and treat people infected with HIV. There are worrying gaps in the provision of these tests, which check HIV status and health, and the WHO warns that it could lead to UN targets on ending HIV being missed. Reasons for the gap include lack of reagents, equipment not being properly installed or maintained, and inadequate staff training. Some programmes may have overly–focused on buying equipment without planning for its optimal use. For example, in Zimbabwe, only 5.6% of patients on HIV treatment in 2015 received regular blood checks to monitor their viral load – the target is 21% – and this was largely due to problems with resource mobilisation, specimen transport and equipment procurement. HIV experts Peter Kilmarx and Raiva Simbi highlight that “strong leadership, resources, planning and management are needed to scale–up laboratory services.” (*BBC*, 25 August 2016)

➢ Douglas Henderson, epidemiologist, died on 19 August 2016, and he will be chiefly remembered for leading the WHO’s smallpox eradication campaign. When he began the campaign, the funds allocated for global eradication – US$ 2.7 million – were not enough to purchase vaccines. However, Dr Henderson realised the futility of attempting to vaccinate everyone against smallpox, and focused on surveillance and containment. Whenever a case of smallpox was identified, a team was dispatched to identify and vaccinate everyone whom that person had been in contact with, and their contacts. He also insisted that policy could only be set by people actively engaged in smallpox fieldwork. Dr Henderson believed that his greatest achievement was the WHO’s expanded program on immunisation, which aimed to provide universal access to necessary immunisations. “I believe that the important, longer–term contribution of smallpox eradication … was its demonstration of how much could be accomplished with how little in the control of infectious diseases through community–wide vaccination programmes,” he wrote in his 2009 autobiography *Small–pox – the Death of a Disease.* (*Guardian*, 7 September 2016)

